# Supplementary material for: The COVID-19 related stress and social network addiction among Chinese college students: A moderated mediation model
Source: PLoS One. 2023 Aug 25;18(8):e0290577. doi: 10.1371/journal.pone.0290577 (PMC10456156; doi:10.1371/journal.pone.0290577)
Supplement: S1 Checklist — (DOCX) [file pone.0290577.s001.docx]

STROBE Statement—checklist of items that should be included in reports of observational studies

|  | Item No. | Recommendation | Page  No. | Relevant text from manuscript |
| --- | --- | --- | --- | --- |
| **Title and abstract** | 1 | (*a*) Indicate the study’s design with a commonly used term in the title or the abstract | 1-2 | A Moderated Mediation Model; A questionnaire survey was conducted |
|  |  | (*b*) Provide in the abstract an informative and balanced summary of what was done and what was found | 2 | Based on social cognitive theory and gender differences, this study verified a moderated mediation model to explore the relationship between the COVID-19 related stress (CRS) and social network addiction (SNA) and evaluate the mediating role of fear of missing out (FoMO) and the moderating role of gender. A questionnaire survey was conducted, including 702 Chinese university students.This study used PROCESS to test the hypothesis model.The results showed that the CRS significantly and positively affected the SNA of college students and FoMO played a complementary mediating role. Moreover, the analysis of the moderated mediation model showed that gender moderated the relationship between FoMO and SNA; the effect of FoMO was stronger on the SNA of male college students than that of females. The results not only enhanced our understanding of the internal influencing mechanism of the relationship between CRS and SNA but also considered gender differences. In addition, some suggestions were proposed. |
| Introduction | | | |  |
| Background/rationale | 2 | Explain the scientific background and rationale for the investigation being reported | 2-7 | as specific details in the manuscript Page 2-7 |
| Objectives | 3 | State specific objectives, including any prespecified hypotheses | 6-10 | hypothesis 1: CRS significantly and positively influences SNA in Chinese college students.  hypothesis 2: FoMO has a mediating role between CRS and SNA.  hypothesis 3: Gender moderates the relationship between CRS, FoMO, and SNA. |
| Methods | | | |  |
| Study design | 4 | Present key elements of study design early in the paper | 11-14 | as specific details in the manuscript Page 11-14 |
| Setting | 5 | Describe the setting, locations, and relevant dates, including periods of recruitment, exposure, follow-up, and data collection | 11-13 | as specific details in the manuscript Page 11-13 |
| Participants | 6 | (*a*) *Cohort study*—Give the eligibility criteria, and the sources and methods of selection of participants. Describe methods of follow-up  *Case-control study*—Give the eligibility criteria, and the sources and methods of case ascertainment and control selection. Give the rationale for the choice of cases and controls  *Cross-sectional study*—Give the eligibility criteria, and the sources and methods of selection of participants | 11-12 | as specific details in the manuscript Page 11-12 |
|  |  | (*b*) *Cohort study*—For matched studies, give matching criteria and number of exposed and unexposed  *Case-control study*—For matched studies, give matching criteria and the number of controls per case |  |  |
| Variables | 7 | Clearly define all outcomes, exposures, predictors, potential confounders, and effect modifiers. Give diagnostic criteria, if applicable | 12-20 | as specific details in the manuscript Page 12-20 |
| Data sources/ measurement | 8* | For each variable of interest, give sources of data and details of methods of assessment (measurement). Describe comparability of assessment methods if there is more than one group | 12-16 | as specific details in the manuscript Page 12-16 |
| Bias | 9 | Describe any efforts to address potential sources of bias | 11 and 16 | In this study, purposive sampling was used to recruit college students at a university in Southern China. The criteria for recruiting participants were college students who voluntarily wanted to participate should be recruited. First of all, professional training was provided to teachers who would distribute the questionnaire. The questionnaire and items were explained to make them understand the purpose of the study. Second, the participants were informed of the anonymous submission of the questionnaires, the study's purpose, and the confidentiality agreement. After obtaining consent from participants, questionnaires were distributed through the online questionnaire platform Questionnaire Star (www.wjx.cn). By scanning a two-dimensional barcode, participants could complete the survey with the help of their teachers. They could refuse or withdraw from the study anytime before submission.  CMV Test |
| Study size | 10 | Explain how the study size was arrived at | 12 | According to the formula for calculating the sample size by Israel [92] , the official sample size of this study should not be less than 652. In our case, 777 questionnaires were distributed, 702 valid questionnaires were returned (75 invalid questionnaires were excluded). The effective rate was 90.35%. |

Continued on next page

| Quantitative variables | 11 | Explain how quantitative variables were handled in the analyses. If applicable, describe which groupings were chosen and why | 13-14 | Statistical analysis |
| --- | --- | --- | --- | --- |
| Statistical methods | 12 | (*a*) Describe all statistical methods, including those used to control for confounding | 13-14 | Statistical analysis |
|  |  | (*b*) Describe any methods used to examine subgroups and interactions |  |  |
|  |  | (*c*) Explain how missing data were addressed |  |  |
|  |  | (*d*) *Cohort study*—If applicable, explain how loss to follow-up was addressed  *Case-control study*—If applicable, explain how matching of cases and controls was addressed  *Cross-sectional study*—If applicable, describe analytical methods taking account of sampling strategy |  |  |
|  |  | (*e*) Describe any sensitivity analyses |  |  |
| Results | | | | |
| Participants | 13* | (a) Report numbers of individuals at each stage of study—eg numbers potentially eligible, examined for eligibility, confirmed eligible, included in the study, completing follow-up, and analysed | 11-12 | as specific details in the manuscript Page 11-12 |
|  |  | (b) Give reasons for non-participation at each stage |  | NA |
|  |  | (c) Consider use of a flow diagram |  | NA |
| Descriptive data | 14* | (a) Give characteristics of study participants (eg demographic, clinical, social) and information on exposures and potential confounders | 11-12 | In our case, 777 questionnaires were distributed, 702 valid questionnaires were returned (75 invalid questionnaires were excluded). The effective rate was 90.35%. There were 170 (24.2%) male and 532 (75.8%) female students; 236 (33.6%) only children and 466 (66.4%) non-only children; 317 (45.2%) first year university students, 160 (22.8%) second year students, 152 (21.7%) third year students, and 30 (4.3%) fourth year students; and 43 master’s students (6.1%). The gender imbalance in the sample is because the sampled university was a normal university, including a larger proportion of female students in the natural sample composition. |
|  |  | (b) Indicate number of participants with missing data for each variable of interest |  |  |
|  |  | (c) *Cohort study*—Summarise follow-up time (eg, average and total amount) |  |  |
| Outcome data | 15* | *Cohort study*—Report numbers of outcome events or summary measures over time |  |  |
|  |  | *Case-control study—*Report numbers in each exposure category, or summary measures of exposure |  |  |
|  |  | *Cross-sectional study—*Report numbers of outcome events or summary measures | 17-20 | as specific details in the manuscript Page 17-20 |
| Main results | 16 | (*a*) Give unadjusted estimates and, if applicable, confounder-adjusted estimates and their precision (eg, 95% confidence interval). Make clear which confounders were adjusted for and why they were included |  |  |
|  |  | (*b*) Report category boundaries when continuous variables were categorized |  |  |
|  |  | (*c*) If relevant, consider translating estimates of relative risk into absolute risk for a meaningful time period |  |  |

Continued on next page

| Other analyses | 17 | Report other analyses done—eg analyses of subgroups and interactions, and sensitivity analyses | 14-20 | as specific details in the manuscript Page 14-20 |
| --- | --- | --- | --- | --- |
| Discussion | | | | |
| Key results | 18 | Summarise key results with reference to study objectives | 20-22 | as specific details in the manuscript Page 20-22 |
| Limitations | 19 | Discuss limitations of the study, taking into account sources of potential bias or imprecision. Discuss both direction and magnitude of any potential bias | 24 | Limitations and future research directions |
| Interpretation | 20 | Give a cautious overall interpretation of results considering objectives, limitations, multiplicity of analyses, results from similar studies, and other relevant evidence | 20-23 | as specific details in the manuscript Page 20-23 |
| Generalisability | 21 | Discuss the generalisability (external validity) of the study results | 20-23 | as specific details in the manuscript Page 20-23 |
| Other information | |  | | |
| Funding | 22 | Give the source of funding and the role of the funders for the present study and, if applicable, for the original study on which the present article is based |  | This research did not receive any specific grant from funding agencies in the public, commercial, or not-for-profit sectors. |

*Give information separately for cases and controls in case-control studies and, if applicable, for exposed and unexposed groups in cohort and cross-sectional studies.

**Note:** An Explanation and Elaboration article discusses each checklist item and gives methodological background and published examples of transparent reporting. The STROBE checklist is best used in conjunction with this article (freely available on the Web sites of PLoS Medicine at http://www.plosmedicine.org/, Annals of Internal Medicine at http://www.annals.org/, and Epidemiology at http://www.epidem.com/). Information on the STROBE Initiative is available at www.strobe-statement.org.
