# Supplementary material for: The COVID-19 related stress and social network addiction among Chinese college students: A moderated mediation model
Source: PLoS One. 2023 Aug 25;18(8):e0290577. doi: 10.1371/journal.pone.0290577 (PMC10456156; doi:10.1371/journal.pone.0290577)
Supplement: Attachment — Submitted filename: Review report.docx [file pone.0290577.s002.docx]

The authors presented a study on the topic: “The COVID-19 Related Stress and Social Network Addiction among Chinese College Students: A Moderated Mediation Model” which examines the relationship between COVID-19 related stress (CRS) and social network addiction (SNA) among Chinese university students. In this study, the authors adopted a social cognitive theory and gender differences as determining factors in their theoretical framework. Both factors investigate the mediating role of fear of missing out (FoMO) as well as the moderating role of gender. There are few comments I wish to address as follows:

1. The hypothetical problem is not very clear; I would suggest that the authors outline their hypothesis by identifying the hypothesis and stating them in order. Authors can use bulletins to do that.
2. Authors should relate their findings according to the stated hypothesis.
3. The tables in the article should be properly drawn. Some lines are hidden with text hovering over the lines.
4. According to the gender influence in the study, can the authors provide an explanation or theoretical justification for why males might be more affected by FoMO compared to females.
