# Supplementary material for: The COVID-19 related stress and social network addiction among Chinese college students: A moderated mediation model
Source: PLoS One. 2023 Aug 25;18(8):e0290577. doi: 10.1371/journal.pone.0290577 (PMC10456156; doi:10.1371/journal.pone.0290577)
Supplement: Attachment — Submitted filename: renamed_da93e.docx [file pone.0290577.s003.docx]

**Response to Reviewer 2 Comments**

Dear reviewer 2:

Thank you very much for your time involved in reviewing the manuscript and your comments have further improved the quality of the manuscript.

We have carefully reviewed the comments and revised the manuscript accordingly. The modified section was already highlighted in yellow. Hope the explanation has fully addressed all of your concerns. Point-by-point response to reviewer are attached below this letter.

Please see the attachment.

**Point 1:** Hypotheses 2 and 3, when speaking of a "mediating or moderating role", it is not clear in what sense. These hypotheses should be stated more concretely.

**Response 1:** Thanks for your advice, we have revised the hypothesis to make the hypothesis reflect more concretely. Please see the lines 166-168, 201-203 and 227-244 of our manuscript for specific modifications.

**Point 2:** In the method, the steps to be followed for the validation of the instruments and the rest of the analysis should be indicated more clearly and in a logical order. The presentation of results should also follow this order, keeping the concordance between each item of the research.

**Response 2:** According to your comments, we have modified the data analysis strategy in the "Statistical analysis" section to correspond to the sequence of results presentation. Please see the lines 295-334 of our manuscript for specific revisions.

**Point 3:** In the practical contributions, the first part emphasizes Covid. A broader context should be given based on the current situation, which is no longer a health emergency. On the other hand, emphasis is placed on the actions to be taken by university teachers, however, the study does not focus on them; in this regard, the practical contributions should be related to the main line of research of this study.

**Response 3:** Thank you for your comments. A broader context in the practice contributions has been given based on your recommendations and we shifted our focus from university teachers to the main line of research of this study (college students ). Please see the lines 523-533 of the manuscript.
